# *Ureaplasma parvum* septic arthritis in LRBA deficiency: An infectious mimic of autoimmune arthritis

**DOI:** 10.70962/jhi.20260082

**Published:** 2026-08-28

**Authors:** Katrien Timmermans, Ciel De Vriendt, Stien Vandendriessche, Lore Pottie, Lisa Roels, Simon J. Tavernier, Filomeen Haerynck, Steven Callens, Leslie Naesens

**Affiliations:** 1 Department of Internal Medicine, Ghent University Hospital, Ghent, Belgium; 2 Department of Internal Medicine and Pediatrics, Ghent University, Ghent, Belgium; 3 Department of Hematology, Ghent University Hospital, Ghent, Belgium; 4 Department of Medical Microbiology, Ghent University Hospital, Ghent, Belgium; 5 Center for Medical Genetics Ghent, Ghent University Hospital, Ghent, Belgium; 6 Department of Biomolecular Medicine, Ghent University, Ghent, Belgium; 7 Primary Immunodeficiency Research Lab, Center for Primary Immunodeficiency, Jeffrey Modell Diagnosis and Research Center, Ghent University Hospital, Ghent, Belgium

## Abstract

We report *Ureaplasma parvum* septic arthritis in lipopolysaccharide-responsive and beige-like anchor protein deficiency during rituximab-based chemotherapy for relapsed Hodgkin lymphoma. This case highlights culture-negative infection mimicking autoimmune arthritis and supports early 16S ribosomal RNA PCR in immunocompromised patients with a primary immune regulatory disorder.

## Teaser

Timmermans et al. report culture-negative septic arthritis caused by *Ureaplasma parvum* in a patient with LRBA deficiency, mimicking autoimmune disease. Using 16S rRNA PCR, they demonstrate the diagnostic value of molecular tools and highlight that humoral immunodeficiencies predispose to invasive infections with fastidious organisms such as *Ureaplasma*.

## Introduction

A broad spectrum of inborn errors of immunity (IEIs) affecting adaptive immunity may present with autoimmunity (1). These patients are also highly susceptible to infections, both intrinsically and due to immunosuppressive therapy, including those caused by fastidious organisms. Consequently, culture-negative infections may be misdiagnosed as autoimmune manifestations in patients with IEI, leading to inappropriate immunosuppressive treatment.

Lipopolysaccharide-responsive and beige-like anchor protein (LRBA) deficiency is a prototypical primary immune regulatory disorder, characterized by impaired immune tolerance, leading to recurrent infections, immune dysregulation, and recurrent polyautoimmunity (2). LRBA regulates cytotoxic T lymphocyte–associated protein 4 (CTLA-4) surface expression, an inhibitory receptor constitutively expressed on regulatory T cells (Tregs) and induced on activated T cells. Initially, LRBA deficiency was classified as common variable immunodeficiency (CVID) with autoimmune complications because of recurrent infections, hypogammaglobulinemia, and impaired B cell maturation. Autoimmunity is one of the most frequent manifestations of LRBA deficiency, reported in 82% of patients in a systematic review, and may include hematologic, gastrointestinal, endocrine, and rheumatologic involvement (2). Arthritis is part of the LRBA phenotype, reported in 18% of evaluated patients, and may resemble juvenile idiopathic arthritis, typically presenting as oligoarticular or polyarticular disease. In addition, LRBA deficiency is associated with an increased risk of malignancy, particularly Epstein-Barr virus (EBV)–associated lymphoproliferative disorders, reflecting impaired immune surveillance.

Here, we describe a patient with LRBA deficiency undergoing treatment for relapsed Hodgkin lymphoma who developed culture-negative septic arthritis caused by *Ureaplasma parvum*, diagnosed by 16S ribosomal RNA (16S rRNA) PCR on synovial fluid. This case underscores the susceptibility of patients with immune dysregulation and treatment-induced humoral immunodeficiency to fastidious infections caused by *Ureaplasma* and *Mycoplasma*, as well as the risk of misdiagnosing these infections as autoimmune disease.

## Case presentation

An 18-year-old Iranian woman was diagnosed with stage IVB Hodgkin lymphoma and treated with six cycles of doxorubicin, bleomycin, vinblastine, and dacarbazine chemotherapy. Posttreatment ^18^F-fluorodeoxyglucose positron emission tomography–computed tomography (^18^F-FDG PET-CT) revealed persistent diffuse lymphadenopathy and splenomegaly. Subsequent lymph node biopsy showed polyclonal follicular hyperplasia, without evidence of lymphoma relapse.

6 months after completion of chemotherapy, peripheral blood immunophenotyping showed marked lymphopenia, with total lymphocytes of 346/μl (1,230–3,420/μl). T cell phenotyping revealed reduced CD3 T cells at 272/μl (700–2,100/μl), CD4 T cells at 164/μl (300–1,400/μl), and CD8 T cells at 93/μl (200–1,200/μl). B cell immunophenotyping showed reduced total B cells at 14/μl (100–500/μl), with markedly decreased non-switched memory B cells at 2.2% and 0.3/μl (4–46% and 11–129/μl, respectively) and switched memory B cells at 1.6% and 0.2/μl (7–33% and 11–98/μl, respectively). Serum immunoglobulin analysis showed mildly increased IgG at 18.1 g/L (7–16 g/L), normal IgM at 0.48 g/L (0.40–2.48 g/L), and mildly reduced IgA at 0.57 g/L (0.71–3.65 g/L).

**Figure 1.**
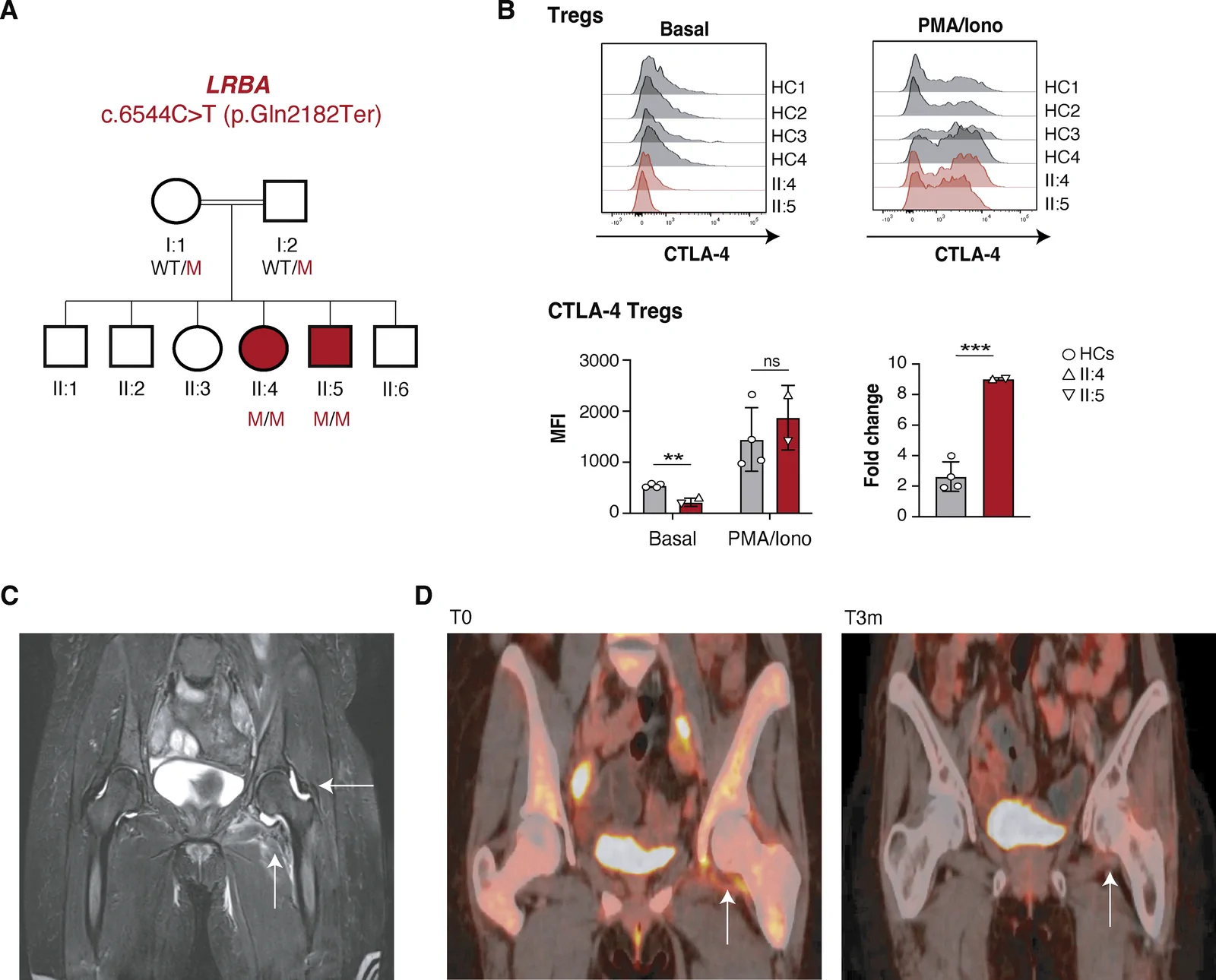
***Ureaplasma parvum* septic arthritis in a patient with LRBA deficiency. (A)** Family pedigree with affected individuals indicated in red. **(B)** Flow cytometric analysis of intracellular CTLA-4 expression in Tregs from affected family members (II:4, II:5) and HCs, under unstimulated conditions and following 2-h stimulation with PMA/ionomycin, according to an optimized in-house protocol. **(C)** MRI demonstrating joint effusion of the femoroacetabular joint (upper arrow) with associated edema of the left adductor muscles (lower arrow). **(D)** Serial ^18^F-FDG PET-CT imaging at diagnosis and after 3 months of treatment, demonstrating metabolic resolution of septic arthritis of the left hip (arrows). Physiological ovarian hypermetabolism is visible on the initial PET-CT. **P < 0.01 and ***P < 0.001 (two-tailed, unpaired Student’s *t* test). ns, statistically not significant. PMA, phorbol myristate acetate; HC, healthy control.

Her younger brother had recurrent immune thrombocytopenia from childhood, for which he received multiple courses of rituximab and corticosteroids. He also exhibited additional features of immune dysregulation, including autoimmune enteropathy and granulomatous lymphocytic interstitial lung disease. Post-rituximab immunophenotyping showed absent peripheral B cells and panhypogammaglobulinemia. At 16 years of age, whole-exome sequencing identified LRBA deficiency caused by a homozygous nonsense variant in exon 42 of 58 (*LRBA* c.6544C>T; p.Gln2182Ter), predicted to result in nonsense-mediated mRNA decay (Fig. 1 A). Targeted Sanger sequencing was subsequently performed in his sister at 20 years of age and confirmed the same homozygous variant. Functional validation demonstrated reduced basal CTLA-4 expression on memory Tregs, which normalized upon stimulation, confirming pathogenicity (Fig. 1 B).

In the sister, progressive fatigue at 23 years of age prompted follow-up ^18^F-FDG PET-CT, which demonstrated increased metabolic activity and enlargement of lymphadenopathies. Repeat lymph node biopsy confirmed relapsed immunodeficiency-associated Hodgkin lymphoma, with EBV positivity and CD20 expression. Salvage therapy with rituximab, dexamethasone, cytarabine, and cisplatin chemotherapy was initiated. Following the second chemotherapy cycle, she developed insidious left hip and groin pain with an antalgic gait. She was subfebrile and had elevated inflammatory markers, with C-reactive protein levels ranging from 50 to 100 mg/L. Ultrasound revealed joint effusion, and aspiration yielded inflammatory synovial fluid containing 19,850 leukocytes/μl, with 73.3% neutrophils and no crystals. Routine bacterial cultures remained negative in the absence of prior antibiotic exposure.

An initial diagnosis of autoimmune or reactive arthritis was considered, given the indolent presentation and underlying LRBA deficiency. Empiric treatment with nonsteroidal anti-inflammatory drugs (diclofenac 75 mg twice daily) and broad-spectrum antibiotics (flucloxacillin 6 g daily and ceftriaxone 2 g daily) led to transient improvement. However, symptoms persisted, with worsening functional impairment, prompting further investigation. Imaging, including magnetic resonance imaging (MRI) and ^18^F-FDG PET-CT, confirmed arthritis of the left hip (Fig. 1, C and D). Repeated aspirations consistently showed inflammatory, culture-negative synovial fluid. Histopathological examination of synovial biopsy tissue demonstrated neutrophilic inflammation and granulation tissue. Broad-range 16S rRNA PCR followed by Sanger sequencing was ultimately performed on synovial fluid and identified *Ureaplasma parvum*, establishing the diagnosis of septic arthritis.

Combination antimicrobial therapy with doxycycline 200 mg once daily and azithromycin 250 mg once daily was initiated empirically in the absence of phenotypic susceptibility data, resulting in marked clinical improvement within 2 wk. As serum IgG levels declined to 2.4 g/L following prior immunochemotherapy, monthly intravenous immunoglobulin replacement therapy was started. Consolidation therapy with carmustine, etoposide, cytarabine, and melphalan conditioning followed by autologous stem cell transplantation was subsequently performed. Given her severe posttransplant immunocompromised state, suppressive antibiotic therapy was continued and remains ongoing after 20 months. In parallel, a donor search is underway in preparation for consolidative allogeneic hematopoietic stem cell transplantation to address her underlying IEI. The patient remains in clinical remission, with resolution of inflammatory activity on ^18^F-FDG PET-CT (Fig. 1 D).

## Discussion

*Ureaplasma* and *Mycoplasma* species belong to the class Mollicutes, among the smallest self-replicating organisms (3). These bacteria evolved from Gram-positive ancestors but lack a peptidoglycan cell wall and have limited biosynthetic capacity, resulting in dependence on host-derived nutrients. Mollicutes commonly colonize the oropharyngeal, respiratory, and urogenital tracts. Clinically relevant species include *Mycoplasma pneumoniae*, *Mycoplasma hominis*, and *Mycoplasma genitalium*, as well as *Ureaplasma parvum* and *Ureaplasma urealyticum*. While typically commensal in the urogenital tract, *Ureaplasma* species may become invasive in the setting of immunosuppression or mucosal disruption. Extra-genitourinary infections are rare but recognized in immunocompromised hosts. Septic arthritis due to *Ureaplasma* has been reported predominantly in patients with hypogammaglobulinemia, including CVID, and those receiving rituximab, highlighting the critical role of humoral immunity in defense against these organisms (4).

In our patient, LRBA deficiency provided the underlying context of immune dysregulation, EBV-associated lymphomagenesis, and need for intensive immunochemotherapy. Susceptibility to opportunistic infection was likely multifactorial, resulting from both LRBA deficiency and treatment-induced immunosuppression. The most direct contributors to susceptibility were probably profound secondary humoral immunodeficiency and cellular immunosuppression following rituximab-containing salvage chemotherapy, with deep hypogammaglobulinemia. Thus, this case illustrates how immune dysregulation predisposes to opportunistic infection through both the genetic defect and therapies used to control disease and associated malignancy.

In patients with immune dysregulation, distinguishing autoimmune from infectious arthritis is essential, as inappropriate immunosuppression may exacerbate occult infection. Imaging findings are often nonspecific and insufficient to differentiate these etiologies. Diagnosis is further complicated by the fact that conventional microbiological cultures frequently fail to detect Mollicutes, while Gram staining is uninformative because these organisms lack a cell wall. Therefore, specialized culture techniques or molecular diagnostics may be required. Molecular diagnostics have emerged as valuable tools for detecting fastidious organisms and should be considered early when an infectious cause is suspected in culture-negative arthritis. In this case, 16S rRNA sequencing was performed using an in-house Sanger sequencing protocol with universal primers. Basic Local Alignment Search Tool analysis on the National Center for Biotechnology Information platform showed an E-value of 0.0, indicating a highly specific match with *Ureaplasma parvum*. Plasma metagenomic sequencing of microbial cell-free DNA may represent an additional molecular diagnostic approach for detecting fastidious organisms in culture-negative arthritis, particularly when invasive sampling is difficult to obtain.

A limitation of molecular diagnostics is the absence of antimicrobial susceptibility data. Mollicutes are intrinsically resistant to cell wall–targeting agents (e.g., β-lactams, glycopeptides). Tetracyclines, macrolides, and fluoroquinolones remain active, although resistance patterns vary geographically and are increasing worldwide (5). Given increasing resistance of *Ureaplasma* species to second-generation fluoroquinolones, macrolides or tetracyclines are generally recommended for treatment. In severe infections, combination therapy may be justified in the absence of susceptibility testing, as was applied in our case. There are currently no evidence-based guidelines for the treatment of *Ureaplasma* septic arthritis. The optimal duration remains undefined and ranges from several weeks to several months. Treatment duration should be individualized based on clinical response and the degree of ongoing immunosuppression.

This case illustrates the diagnostic challenge of culture-negative septic arthritis caused by fastidious pathogens in patients with IEIs characterized by immune dysregulation. The risk of invasive *Ureaplasma* infection may arise less from the primary genetic defect itself than from disease-related complications and rituximab-based immunosuppressive therapy, which can lead to profound secondary humoral immunodeficiency. Clinically, *Ureaplasma* septic arthritis may mimic autoimmune arthritis, potentially delaying appropriate antimicrobial treatment. Early use of molecular diagnostics, such as 16S rRNA PCR on synovial fluid, is therefore crucial for accurate diagnosis and timely management of culture-negative arthritis in immunocompromised patients.

## Consent statement

Clinical data and patient samples were collected with informed consent from all participants, in accordance with the Declaration of Helsinki (1975) and local regulations. Informed consent was obtained from the participant/patient(s) for the publication of this case report.

## Data Availability

The data are available from the corresponding author upon request. The disease-causing genetic variant has been deposited in ClinVar under accession number VCV004871841.1.
